# Characterisation of exacerbation risk and exacerbator phenotypes in the POET-COPD trial

**DOI:** 10.1186/1465-9921-14-116

**Published:** 2013-10-29

**Authors:** Kai M Beeh, Thomas Glaab, Susanne Stowasser, Hendrik Schmidt, Leonardo M Fabbri, Klaus F Rabe, Claus F Vogelmeier

**Affiliations:** 1 insaf Respiratory Research Institute, Wiesbaden, Germany; 2 Boehringer Ingelheim Pharma GmbH & Co. KG, Ingelheim am Rhein, Germany; 3 Section of Respiratory Diseases, Department of Medical and Surgical Specialties, University of Modena & Reggio Emilia, Modena, Italy; 4 Christian Albrechts University Kiel, Lung Clinic Grosshansdorf, Grosshansdorf, Hamburg, Germany; 5 University Medical Center Giessen and Marburg, Philipps-Universität Marburg, Baldingerstrasse, D 35043 Marburg, Germany

**Keywords:** Chronic obstructive pulmonary disease, Exacerbations, Mortality, Hospitalisation, Tiotropium, Salmeterol, GOLD

## Abstract

**Background:**

Data examining the characteristics of patients with frequent exacerbations of chronic obstructive pulmonary disease (COPD) and associated hospitalisations and mortality are scarce.

**Methods:**

*Post*-*hoc* analysis of the Prevention Of Exacerbations with Tiotropium in COPD (POET-COPD) trial, targeting exacerbations as the primary endpoint. Patients were classified as non-, infrequent, and frequent exacerbators (0, 1, or ≥ 2 exacerbations during study treatment), irrespective of study treatment. A multivariate Cox regression model assessed the effect of covariates on time to first exacerbation.

**Results:**

In total, 7376 patients were included in the analysis: 63.5% non-exacerbators, 22.9% infrequent, 13.6% frequent exacerbators. Factors significantly associated with exacerbation risk were age, sex, body mass index, COPD duration and severity, smoking history, baseline inhaled corticosteroid use, and preceding antibiotic or systemic corticosteroid courses. Frequent exacerbators had greater severity and duration of COPD, received more pulmonary medication, and ≥ 2 systemic corticosteroid or antibiotic courses in the preceding year, and were more likely to be female and ex-smokers. The small proportion of frequent exacerbators (13.6%) accounted for 56.6% of exacerbation-related hospitalisations, which, overall, were associated with a three-fold increase in mortality.

**Conclusion:**

The frequent exacerbator phenotype was closely associated with exacerbation-related hospitalisations, and exacerbation-related hospitalisations were associated with poorer survival.

**Trial registration:**

NCT00563381; Study identifier: BI 205.389.

## Background

Exacerbations are an important element of the natural course of chronic obstructive pulmonary disease (COPD) and a key component of disease assessment, both in the clinic and as an outcome measure in clinical trials [1-3]. Exacerbations impact disease progression, quality of life and prognosis [4-6].

Therefore, the 2011 revision and 2013 update of the Global Initiative for Chronic Obstructive Lung Disease (GOLD) recommendations highlight the important influence of exacerbations on future risk in COPD, making their prevention a priority [3,7]. In a large observational cohort study, a history of COPD exacerbations was the most reliable predictor of future events, regardless of the severity of airflow limitation [8]. However, COPD is a heterogeneous disease [9,10], with considerable variability in exacerbation susceptibility between patients. The term 'frequent exacerbator’ has been proposed to describe a distinct and stable COPD phenotype at high risk of recurrent events [8,11]. Although these studies were not specifically designed to detect exacerbations of COPD, identifying factors associated with COPD exacerbation risk remains an important aspect of patient monitoring and may help to guide the use of preventative interventions.

The 1-year Prevention Of Exacerbations with Tiotropium in COPD (POET-COPD) study complements and extends the few previous reports on exacerbation phenotypes. This trial, being a large randomised trial using exacerbations as the primary endpoint, enables us to study more than 4400 moderate or severe exacerbations [12]. The purpose of this *post*-*hoc* analysis of the POET-COPD trial was to: determine the factors contributing to exacerbation risk; evaluate the characteristics of patients with infrequent, frequent and severe exacerbations in a large patient population; and, assess the effects of exacerbations on the incidence of hospitalisations and mortality.

## Methods

### Study design

POET-COPD (ClinicalTrials.gov number: NCT00563381) was a prospective, 1-year, randomised, double-blind, double-dummy, parallel-group study comparing effects of tiotropium (18 μg once daily via HandiHaler^®^) and salmeterol (50 μg twice daily via pressurised metered-dose inhaler) on moderate and severe COPD exacerbations. The study, carried out in 725 centres in 25 predominantly European countries from January 2008 until April 2010, was conducted in accordance with the Declaration of Helsinki (1996) and Good Clinical Practice. Ethical approval was obtained from independent ethics committees or institutional review boards and all patients provided written informed consent. Details of the study protocol and main results have been described previously [12,13].

### Patients

Briefly, patients were eligible for study inclusion if they were aged ≥ 40 years, had a smoking history of ≥ 10 pack-years, and a diagnosis of moderate to very severe COPD with a post-bronchodilator forced expiratory volume in 1 second (FEV_1_) of ≤ 70% of the predicted value, and FEV_1_/forced vital capacity ratio of ≤ 70% [12]. A documented history of ≥ 1 exacerbation treated with systemic glucocorticoid or antibiotic therapy, or hospitalisation within the previous year, was required. The main exclusion criteria were a diagnosis of asthma, severe cardiovascular disorders, moderate or severe renal insufficiency (creatinine clearance ≤ 50 ml/min) and significant diseases other than COPD that in the opinion of the Investigator may put the patient at risk or may influence either the results of the study or the patient’s ability to participate in the trial. The use of systemic corticosteroid medication at unstable doses or at doses in excess of the equivalent of 10 mg prednisolone per day or 20 mg every other day was also excluded. Full details of exclusion criteria have been reported previously [12,13]. Participation in a pulmonary rehabilitation programme was not an exclusion criterion in the trial. Throughout the study, patients were permitted to continue previously prescribed COPD treatments (including stable systemic corticosteroids up to 10 mg prednisolone or equivalent daily); only anticholinergics and β_2_-agonists other than salbutamol for rescue medication were prohibited.

### Assessments

The primary endpoint of POET-COPD was time to first COPD exacerbation. Secondary exacerbation endpoints included a variety of time-to-event and number-of-event outcomes. An exacerbation was defined as an increase in, or new onset of, ≥ 2 respiratory symptoms (cough, sputum, dyspnoea, wheezing, chest tightness) with ≥ 1 symptom lasting ≥ 3 days and leading the patient’s attending physician to initiate treatment with systemic corticosteroids and/or antibiotics (moderate exacerbation) or hospitalisation (severe exacerbation).

Data on the occurrence of exacerbations and their treatment were collected prospectively for all patients while on study treatment via case report forms that were administered during scheduled clinic visits (at Months 2, 4, 8 and 12), and by monthly telephone contact between these visits. The end of an exacerbation was determined by the investigator; a recurrent exacerbation was defined as an event that occurred ≥ 7 days after the preceding exacerbation.

### Statistical analysis

All randomised patients who received at least one dose of study medication were classified, irrespective of study drug allocation, according to their exacerbation frequency into 'non-exacerbators’, 'infrequent exacerbators’ and 'frequent exacerbators’ if they had experienced 0, 1 or ≥ 2 exacerbations, respectively, while on study treatment.

Descriptive statistical methods, including exposure-adjusted crude incidence rates, were applied to characterise non-, infrequent, or frequent exacerbators as well as those patients who experienced ≥ 1 severe exacerbation.

A multivariate Cox regression model was developed for time to first COPD exacerbation, using the stepwise selection procedure to assess the association between baseline covariates and exacerbation risk. This selection procedure consisted of a series of alternating forward selection and backward elimination steps [14].

A covariate had to be significant at the 0.25 level before being entered into the model and significant at the 0.15 level to remain in the model. Study treatment was an additional covariate in the multivariate model to account for its known effect on time to first exacerbation. Overall p-values were calculated using Wald’s χ^2^ test for optimal model.

Sensitivity analyses of the multivariate Cox regression model selection were performed using both backward selection and completer analysis. Robustness of the model selection was checked via the bootstrapping technique based on 1000 repetitions of the selection procedure. Moreover, both baseline characterisation and multivariate Cox regression were performed on data from the subgroup of patients who completed planned study treatment. All statistical analyses were performed using SAS^®^ software, version 9.2 (SAS Institute Inc, Cary, NC, USA).

## Results

### Patients

In total, 7376 patients received at least one dose of study medication. There were 4411 COPD exacerbation episodes among 2691 (36.5%) patients, and 725 severe exacerbations among 598 (8.1%) patients. Of all exacerbations, 23% (1004/4411) were followed by ≥ 1 recurrent exacerbation and 39% of all exacerbations (1720/4411) were recurrent exacerbations.

### Covariates associated with exacerbation risk

**Figure 1:**
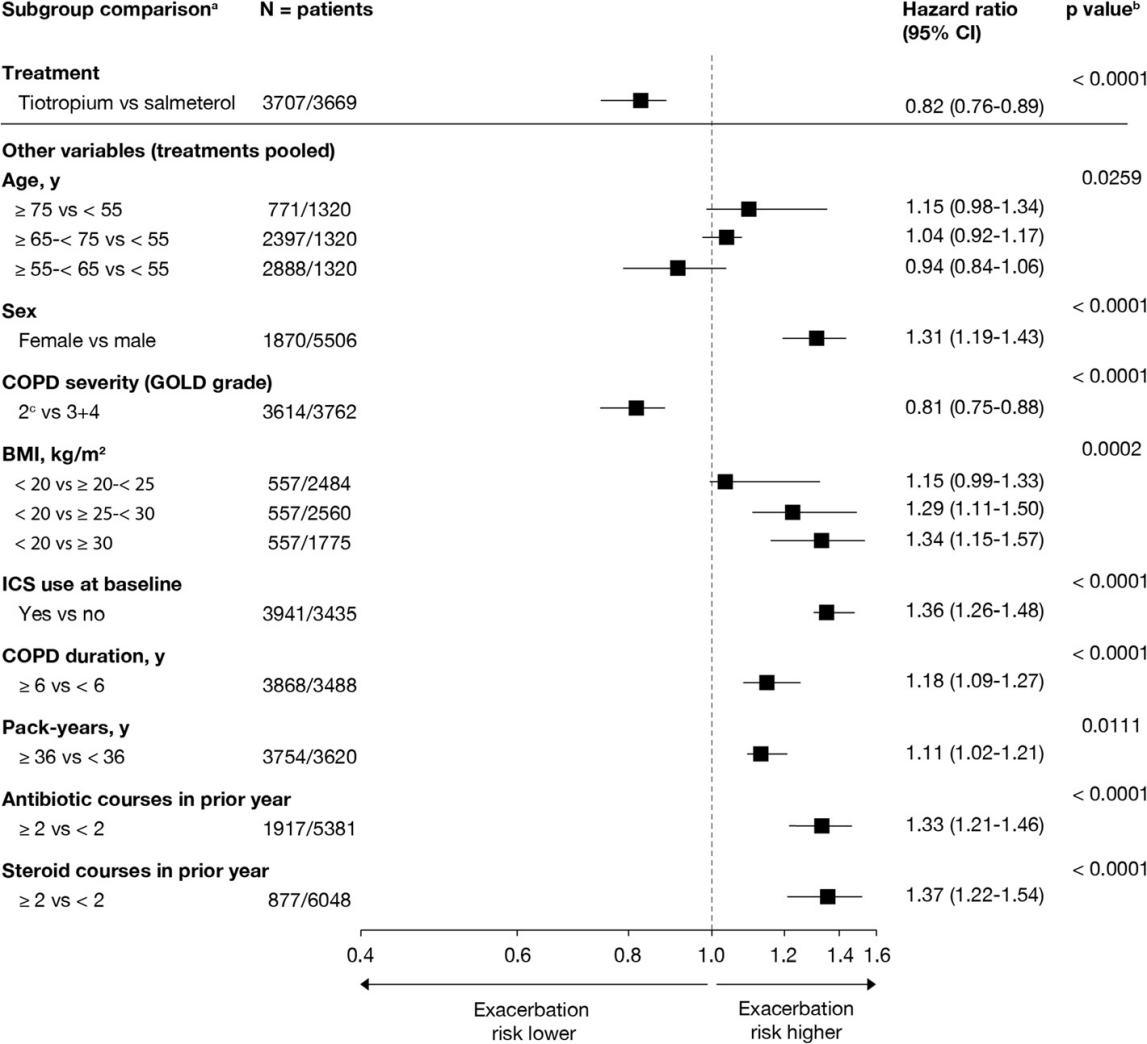
**Multivariate model for time to first COPD exacerbation (all) and effect of covariates.** Time to first COPD exacerbation was analysed using multivariate Cox regression with stepwise model selection. Potential covariates originally also included 'smoking status’ with categories 'non-current smoker’; 'current smoker’ and 'concomitant diagnoses at baseline’ with categories '0, 1 and > 1’ but turned out to be non-significant for the significance level chosen. ^a^≥ 1 exacerbation versus no exacerbation. ^b^Overall p-value based on Wald’s χ^2^ test for optimal model. ^c^Includes 23 GOLD category 1 patients. *BMI* = body mass index; *CI* = confidence interval; *COPD* = chronic obstructive pulmonary disease; *GOLD* = Global Initiative for Chronic Obstructive Lung Disease; *ICS* = inhaled corticosteroids.

The Forest plot in Figure 1 shows the results of the stepwise model selection for time to first COPD exacerbation (moderate or severe) using multivariate Cox regression. Baseline covariates identified as potential predictors for exacerbation risk were age, sex, COPD severity (by GOLD category), body mass index (BMI), inhaled corticosteroid (ICS) use at baseline, COPD duration, smoking history (pack-years) and the extent of antibiotic or oral corticosteroid use due to breathing problems in the previous year. The covariates 'smoking status’ and 'concomitant diagnoses at baseline’ were non-significant (p > 0.4). Robustness of the model selection was confirmed by the bootstrapping technique. The risk of a first exacerbation was higher for women compared with men (hazard ratio [HR], 1.31; 95% confidence interval [CI], 1.19 to 1.43); for patients with a mean baseline BMI < 20 kg/m^2^ compared with BMI ≥ 25 to < 30 kg/m^2^ (HR, 1.29; 95% CI, 1.11 to 1.50) or ≥ 30 kg/m^2^ (HR, 1.34; 95% CI, 1.15 to 1.57); patients with ICS use at baseline versus no ICS use (HR, 1.36; 95% CI, 1.26 to 1.48); patients on ≥ 2 antibiotic courses versus < 2 antibiotic courses (HR, 1.33; 95% CI, 1.21 to 1.46); and patients on ≥ 2 systemic corticosteroid courses versus < 2 corticosteroid courses due to breathing problems in the preceding year (HR, 1.37; 95% CI, 1.22 to 1.54), respectively. Sensitivity analyses confirmed these findings (Additional file 1: Table S1).

**Figure 2:**
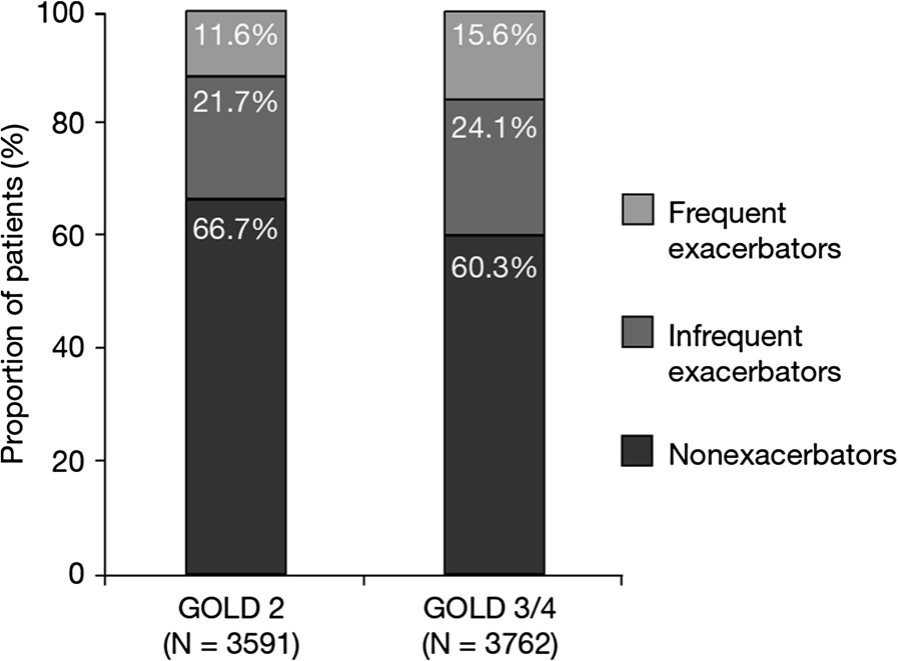
**Exacerbation frequency and severity of airflow limitation (GOLD category).** GOLD category 2 includes 23 patients with GOLD category 1. *GOLD* = Global Initiative for Chronic Obstructive Lung Disease.

Though patients with moderate airflow limitation (GOLD category 2) were associated with a lower exacerbation risk than those at GOLD category 3/4, 11.6% (416/3591) of patients in GOLD category 2 still experienced ≥ 2 exacerbations during the study period compared with 15.6% (588/3762) of patients with severe or very severe airflow limitation (GOLD category 3 and 4) (Figure 2).

### Frequent and severe exacerbation characteristics

**Figure 3:**
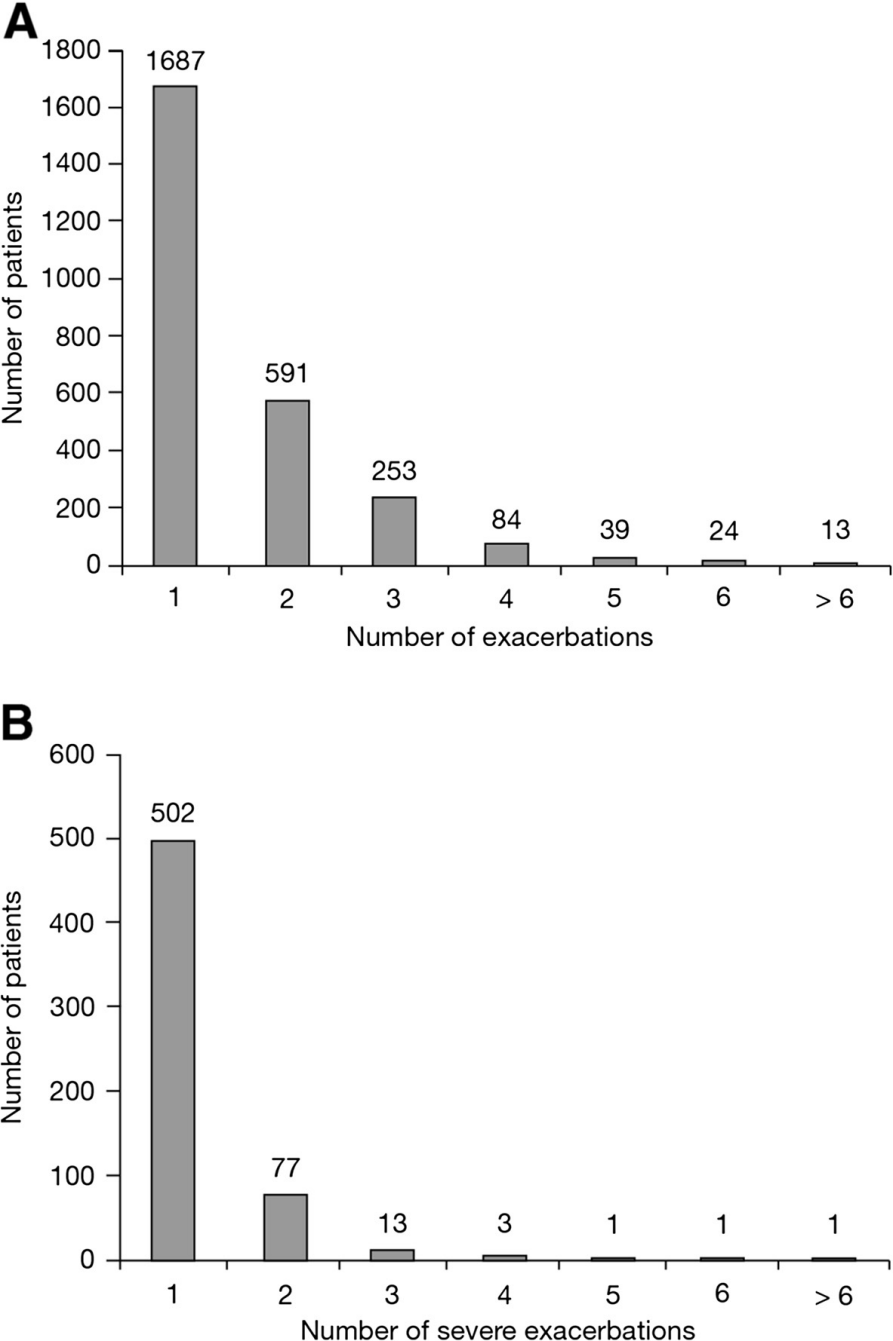
**Frequency distribution of patients with exacerbations by number of (A) exacerbations and (B) severe exacerbations.**

The frequency of exacerbations and exacerbation-related hospitalisations is shown in Figure 3A and 3B. The distributions are highly skewed, with most patients experiencing ≤ 1 moderate or severe exacerbation and only a minority of patients exhibiting a higher frequency of events during study treatment. Based on these data, 4685 (63.5%) patients were categorised as non-exacerbators, 1687 (22.9%) as infrequent and 1004 (13.6%) as frequent exacerbators; 598 patients (8.1%) were categorised as severe exacerbators. The demographics and baseline characteristics for these patient categories are shown in Table 1.

**Table 1:** Demographics and baseline characteristics

	**Non-exacerbators**	**Infrequent exacerbators**	**Frequent exacerbators**	**Severe exacerbators**
	**(n = 4685)**	**(n = 1687)**	**(n = 1004)**	**(n = 598)**
Male sex, %	76.4	72.1	70.9	82.1
Age, years^a^	62.8 (9.0)	63.0 (9.2)	63.0 (8.8)	64.2 (8.8)
BMI, kg/m^2a^	26.8 (5.0)	26.4 (5.3)	26.4 (5.2)	25.8 (5.6)
COPD duration, years^a^	7.6 (6.7)	8.2 (6.5)	8.8 (6.7)	9.1 (6.9)
Current smokers, %	48.8	50.3	41.7	44.6
Smoking history, pack-years^a^	38.1 (19.3)	38.2 (19.4)	39.4 (21.3)	40.3 (20.5)
Post-bronchodilator FEV_1_, L^a^	1.45 (0.46)	1.36 (0.45)	1.32 (0.42)	1.22 (0.42)
Post-bronchodilator FEV_1_, % predicted^a^	50.1 (13.3)	48.2 (13.3)	47.2 (12.6)	43.0 (13.0)
**GOLD category, %**				
2^b^	51.6	46.2	41.4	26.6
3/4	48.4	53.8	58.6	73.4
≥ 2 antibiotic courses in prior year, %^c^	22.6	28.1	38.0	34.8
≥ 2 systemic corticosteroid courses in prior year, %^c^	9.2	13.5	21.9	23.0
**Pulmonary treatment at baseline**				
Tiotropium, %	27.2	35.4	37.0	36.1
LABAs, total/monotherapy, %	48.1 / 8.5	55.1 / 7.6	61.6 / 7.1	54.8 / 5.4
ICS, total/monotherapy, %	49.5 / 9.9	58.0 / 10.6	64.2 / 9.8	63.7 / 14.2
LABA + ICS, %	39.6	47.5	54.5	49.5
Oral corticosteroids, %	2.0	2.8	3.4	3.5
Xanthines, %	20.2	24.4	26.9	28.4
Oxygen, %	0.6	1.2	0.8	1.5

^a^Values are means (SD) if not stated otherwise; ^b^23 patients with GOLD category 1 COPD were included; ^c^Due to breathing problems.

The proportions of premature patient withdrawals were 16.9%, 18.3%, 13.0% and 13.2% in the non-, infrequent, frequent and severe exacerbator subgroups, respectively. Median exposure to study medication in all randomised and treated patients was 361 days in severe exacerbators and 363 days in the other groups.

*BMI* = body mass index; *COPD* = chronic obstructive pulmonary disease; *FEV*_1_ = forced expiratory volume in 1 second; *GOLD* = Global Initiative for Chronic Obstructive Lung Disease; *ICS* = inhaled corticosteroids; *LABA* = long-acting β_2_-agonist; *SD* = standard deviation.

Significant differences in baseline characteristics between patient subgroups were observed for all characteristics except for age and pack-years of smoking. Frequent exacerbators were more likely than non- or infrequent exacerbators to have a longer duration of COPD (p < 0.0001), more severe airflow limitation as assessed by FEV_1_ measures or GOLD category (p < 0.0001), and to have received pulmonary medication at baseline (p < 0.0001 for each drug class; p = 0.0094 for oxygen) or ≥ 2 courses of systemic corticosteroids or antibiotics due to breathing problems in the preceding year (p < 0.0001) and were more likely to be female (p < 0.0001) or ex-smokers (p < 0.0001). Patients with severe exacerbations differed only slightly in their baseline conditions from frequent exacerbators except for severely impaired lung function and a higher prevalence of male subjects (Table 1).

Similar patterns were observed in the sensitivity analyses of patients who completed the protocol-defined treatment period (Additional file 1: Table S2). Treatment of first exacerbations was similar in infrequent and frequent exacerbators (Table 2). Of 1330 subjects treated solely with antibiotics for their first exacerbation, 35% suffered from ≥ 1 subsequent exacerbation; while 1353 subjects who had their first exacerbation treated with systemic corticosteroids or systemic corticosteroids plus antibiotics, 39.7% suffered from ≥ 1 subsequent exacerbation.

**Table 2:** Treatment of first exacerbations in the infrequent and frequent exacerbator subgroups

**Treatment of first exacerbation, n (%)**	**Infrequent exacerbators**	**Frequent exacerbators**
	**(n = 1687)**	**(n= 1004)**
With antibiotics^a^	1481 (87.8)	827 (82.5)
With systemic corticosteroids^b^	816 (48.5)	537 (53.3)
With antibiotics and systemic corticosteroids	615 (36.5)	361 (36.0)
Hospitalisation	315 (18.7)	125 (12.5)

^a^Refers to n = 1686 for infrequent and n = 1003 for frequent exacerbators; ^b^Refers to n = 1684 for infrequent and n = 1004 for frequent exacerbators.

### Exacerbation-related hospitalisations and mortality

The frequent exacerbator group (13.6% of the total study population of 7376 patients) accounted for 56.6% (410/725) of all hospitalisations due to exacerbations (severe exacerbations) during the trial. This corresponds to a crude exposure-adjusted rate of hospitalisations that was twice as high for frequent exacerbators compared with infrequent exacerbators (0.44 vs 0.21 hospitalised exacerbations per patient-year). The proportion of patients with ≥ 1 severe exacerbation was 28.2% in the group of frequent exacerbators and 18.7% in the group of infrequent exacerbators.

**Figure 4:**
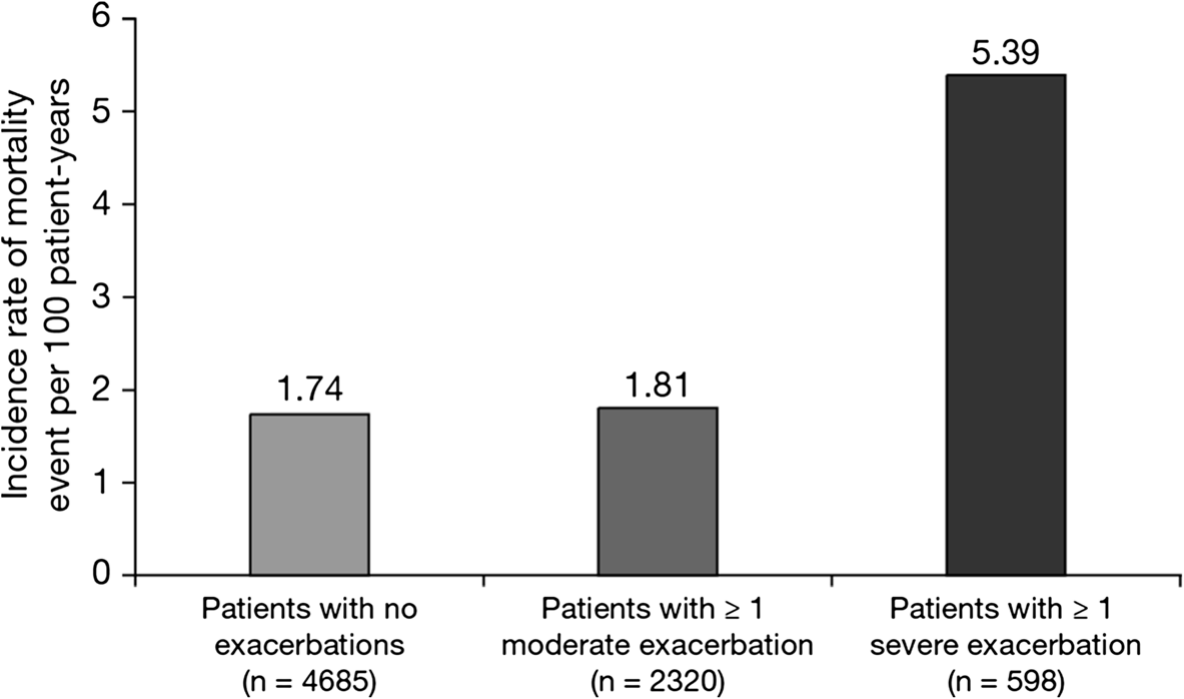
**Incidence rates of all-cause mortality in patients with no exacerbations, patients ≥ 1 moderate exacerbation, and patients experiencing ≥ 1 severe exacerbation while on study treatment.**

Patients with frequent exacerbations differed only slightly from non- or infrequent exacerbators with regard to all-cause mortality (vital status follow-up was complete for 99.1% of all randomised and treated patients): the incidence rates of fatal adverse events (AEs) (onset during planned treatment and date of death up to Day 360) were 1.74, 2.68 and 1.94 per 100 patient-years for non-, infrequent and frequent exacerbators, respectively. However, patients with severe exacerbations had an exposure-adjusted incidence rate of fatal AEs that was approximately three times higher than that of patients without severe exacerbations (incidence rates 1.69, 5.40 and 5.37 per 100 patient-years for patients with 0, 1 or ≥ 2 severe exacerbations, respectively) (Figure 4).

## Discussion

The present findings confirm, contrast, and extend previous investigations of exacerbation phenotypes in patients with COPD. Firstly, it has been confirmed that the risk for developing a frequent or severe exacerbation is largely related to COPD severity, but also affects patients with moderate disease. Secondly, it has been demonstrated that frequent exacerbators accounted for the majority of exacerbation-related hospitalisations, thus establishing a link between frequency and severity of exacerbations. Finally, it has been found that severe exacerbations were associated with a distinct increase in mortality.

The baseline covariates associated with exacerbation risk in our analysis included age, sex, GOLD category, BMI, the use of concomitant ICS at baseline, COPD duration, smoking history (pack-years) and the number of previous courses of antibiotics or systemic corticosteroids, thus reflecting similar findings from previous trials using exacerbations as secondary endpoints [15-17].

To characterise 'high-risk’ patients with frequent COPD exacerbations, we categorised patients into non-, infrequent and frequent exacerbator subgroups. The patients with frequent exacerbations tended to have more severe disease, more pulmonary medication at baseline, and treatment with ≥ 2 antibiotic or ≥ 2 systemic corticosteroid courses in the previous year. Severe exacerbations particularly affected male patients and those with severely impaired airflow limitation.

It is important to note that frequent or severe exacerbations were not restricted to patients with severe airflow limitation (GOLD category 3/4), but also occurred in a significant proportion of patients with moderate COPD (GOLD category 2), despite permitted co-medication and active study treatment [18,19]. The clinical implications of these findings are important, not least because patients with GOLD category 2 disease account for almost half of the COPD patient population [20].

Compared with the Evaluation of COPD Longitudinally to Identify Predictive Surrogate Endpoints (ECLIPSE) study, the proportion of patients categorised as frequent exacerbators was considerably lower in our patient population during the first year. Several reasons may account for these differences, including a higher proportion of patients with severe COPD in the ECLIPSE study and, in particular, using different criteria for defining exacerbations. In POET-COPD, pre-defined criteria needed to be met for an event to be documented as a moderate or severe exacerbation, while ECLIPSE had no such criteria [8,12].

Another intriguing finding is the fact that a minority of patients with frequent exacerbations accounted for the majority of severe exacerbations. In addition, the present analysis builds on previous reports linking severe exacerbations to mortality [4,21,22]. Accordingly, we found an incidence of mortality that was about three times higher in patients with severe exacerbations, regardless of event frequency. This observation, from a large prospective exacerbation trial, supports recent GOLD recommendations that even a single severe exacerbation indicates a high risk of disease progression and poor outcome [3]. We could not confirm recent observations by Lange *et al*. reporting poorer survival in frequent exacerbators [22]. However, such investigations certainly require longer-term follow-up.

Some limitations to the present study must be noted, including the *post*-*hoc* nature of the analysis. In addition, POET-COPD was a pharmacological intervention trial rather than an epidemiological study, and the two treatment interventions differed with respect to the primary outcome. However, by including active study treatment in our multivariate model, this potential source of difference was accounted for. Furthermore, the categorisation into frequent and infrequent exacerbators was based on actual treatment time, without performing a systematic follow-up of exacerbations after premature withdrawal. The higher premature withdrawal rate in non- and infrequent exacerbators may have prevented some of these subjects from being subsequently re-categorised as frequent exacerbators. However, we assume that this did not bias our conclusions given the comparable median exposure ranges in the subgroups, as well as the supportive results from the sensitivity analyses that were restricted to patients who completed the protocol-defined study treatment. Whether the exclusion of high-risk patients may have altered the risk factors for the frequency or severity of exacerbations requires further study. It should be recognised, however, that some of the exclusion criteria were required for consistency with approved warnings and precautions associated with the use of study medications. In POET-COPD, as in most other COPD trials, patients with a history of asthma were excluded from trial participation. This meant that patients with a COPD-asthma overlap, which has been linked to the occurrence of more frequent and severe respiratory exacerbations, were not considered in our analysis [23]. However, it must be noted that there is still much controversy about the designation of patients that share clinical characteristics of COPD and asthma and final validation and clear diagnostic criteria for this joint phenotype must be still developed and prospectively validated [24]. It should also be recognised that patients with characteristic cardiovascular conditions were represented in the study population. Nevertheless, including high-risk patients, particularly with cardiac illnesses, remains a challenging issue for COPD trials [25].

## Conclusions

Our analysis of a large COPD exacerbation trial shows that patients with COPD with a frequent exacerbator phenotype are more likely to be hospitalised. It remains to be determined whether the frequent exacerbator phenotype is a stable condition or varies over time depending on the severity of exacerbations. There is also the need to prospectively investigate the impact of co-morbidities, e.g. cardiovascular, on the frequency and severity of exacerbations of COPD. The fact that severe exacerbations were associated with impaired survival should prompt special awareness to the prevention of any such events.

## Abbreviations

AE: Adverse event; BMI: Body mass index; CI: Confidence interval; COPD: Chronic obstructive pulmonary disease; ECLIPSE: Evaluation of COPD Longitudinally to Identify Predictive Surrogate Endpoints; FEV1: Forced expiratory volume in 1 second; GOLD: Global Initiative for Chronic Obstructive Lung Disease; HR: Hazard ratio; ICS: Inhaled corticosteroids; LABA: Long-acting β_2_-agonist; POET-COPD^®^: Prevention Of Exacerbations with Tiotropium in COPD; SD: Standard deviation.

## Competing interests

Dr Beeh has consulted for Boehringer Ingelheim Pharma GmbH & Co KG, and his institution has received grants from: Almirall, SA; Boehringer Ingelheim Pharma GmbH & Co KG; Cytos Biotechnology; GlaxoSmithKline PLC; Mundipharma International; Novartis AG; Pfizer Inc; and Revotar. Drs Glaab, Stowasser and Schmidt are employees of Boehringer Ingelheim Pharma GmbH & Co KG. Dr Fabbri has consulted for and received lecture fees from: AstraZeneca; Boehringer Ingelheim Pharma GmbH & Co KG; Chiesi Ltd; GlaxoSmithKline PLC; Merck Sharp & Dohme Corp; Novartis AG; Takeda Pharmaceuticals International GmbH; Pfizer Inc; and Sigma-Tau Pharmaceuticals, Inc. Dr Fabbri has also received grants from: AstraZeneca; Boehringer Ingelheim Pharma GmbH & Co KG; Menarini; Merck Sharp & Dohme Corp; Chiesi Ltd; GlaxoSmithKline PLC; Takeda Pharmaceuticals International GmbH; UCB SA, Schering-Plough (now Merck Sharp & Dohme Corp); Pfizer, Inc; and Sigma-Tau Pharmaceuticals, Inc. Dr Rabe has consulted for, participated in advisory board meetings with and received lecture fees from: AstraZeneca; Boehringer Ingelheim Pharma GmbH & Co KG; Chiesi Ltd; Pfizer Inc; Novartis AG; Takeda Pharmaceuticals International GmbH; Merck Sharp & Dohme Corp; and GlaxoSmithKline PLC. Dr Rabe has also received grants from: Novartis AG; AstraZeneca; Boehringer Ingelheim Pharma GmbH & Co KG; Hoffmann-La Roche Inc; Altana; and GlaxoSmithKline PLC. Dr Vogelmeier has consulted for AstraZeneca; Boehringer Ingelheim Pharma GmbH & Co KG; GlaxoSmithKline PLC; Novartis AG; Takeda Pharmaceuticals International GmbH; and Mundipharma International and has received lecture fees from AstraZeneca, Boehringer Ingelheim Pharma GmbH & Co KG, GlaxoSmithKline PLC, Novartis AG, Takeda Pharmaceuticals International GmbH, Talecris Biotherapeutics, Merck Sharp & Dohme Corp and Janssen Global Services, LLC. Dr Vogelmeier has also received grants from Talecris Biotherapeutics.

## Authors’ contributions

KMB contributed to the study design, analysis and interpretation of the results, as well as drafting the manuscript and its subsequent revision. SS contributed to the analysis and interpretation of the results and manuscript drafting and revision. TG contributed to the study idea and design, analysis and interpretation of the results, as well as the manuscript drafting and revision. HS contributed to the study design, statistical analysis and interpretation of the results, as well as the manuscript drafting and revision. LMF and KFR contributed to the analysis and interpretation of the results, as well as the manuscript drafting and revision. CFV contributed to the study idea and design, analysis and interpretation of the results, as well as the manuscript drafting and revision. All authors read and approved the final manuscript.

## Role of the sponsors

Boehringer Ingelheim Pharma GmbH & Co KG was involved in the design of the study, the collection and analysis of the data and in the preparation of the manuscript. Pfizer Inc had no role in the design of the study, collection and analysis of data, or in the preparation of the manuscript.

## Supplementary Material

Additional file 1: Table S1 Demographics and baseline characteristics (completers only). **Table S2.** Multivariate model for time-to-first exacerbation (all) and effect of covariates (completers only). Click here for file
