# Supplementary material for: Characterisation of exacerbation risk and exacerbator phenotypes in the POET-COPD trial
Source: Respir Res. 2013 Oct 29;14(1):116. doi: 10.1186/1465-9921-14-116 (PMC3833311; doi:10.1186/1465-9921-14-116)
Supplement: Additional file 1: Table S1 — Demographics and baseline characteristics (completers only). Table S2. Multivariate model for time-to-first exacerbation (all) and effect of covariates (completers only). [file 1465-9921-14-116-S1.docx]

**Supplementary Material**

**Supplementary Table 1** **Demographics and baseline characteristics (completers only)**

|  | **Nonexacerbators  (n = 3892)** | **Infrequent exacerbators  (n = 1378)** | **Frequent exacerbators  (n = 873)** | **Severe exacerbators**  **(n = 435)** |
| --- | --- | --- | --- | --- |
| Male sex, % | 76.7 | 72.0 | 70.8 | 82.5 |
| Age, y^a^ | 62.5 (8.9) | 62.5 (9.1) | 62.7 (8.9) | 63.3 (8.9) |
| BMI, kg/m^2a^ | 26.9 (5.0) | 26.6 (5.3) | 26.4 (5.1) | 26.1 (5.6) |
| COPD duration, y^a^ | 7.6 (6.6) | 8.1 (6.5) | 8.7 (6.7) | 9.0 (6.9) |
| Current smokers, % | 49.7 | 51.3 | 41.6 | 45.7 |
| Smoked pack-years^a^ | 37.6 (18.8) | 37.4 (19.1) | 38.8 (20.6) | 39.7 (20.6) |
| Postbronchodilator FEV_1_, L^a^ | 1.46 (0.46) | 1.39 (0.46) | 1.33 (0.42) | 1.25 (0.43) |
| Postbronchodilator FEV_1_, % predicted^a^ | 50.2 (12.9) | 49.0 (13.1) | 47.4 (12.6) | 43.3 (12.9) |
| GOLD category, %  2^b^  3/4 | 52.4  47.6 | 48.4  51.6 | 42.0  58.0 | 26.9  73.1 |
| ≥ 2 antibiotic courses in prior year, %^c^  ≥ 2 corticosteroid courses in prior year, %^c^ | 22.0  8.8 | 28.4  12.5 | 38.5  21.0 | 37.0  22.8 |
| Pulmonary treatment at baseline  Tiotropium, %  LABAs, total/monotherapy, %^d^  ICS, total/monotherapy, %^e^  LABA+ICS, %  Oral corticosteroids, %  Xanthines, %  Oxygen, % | 25.8  46.4 / 8.7  47.9 / 10.2  37.7  1.7  20.0  0.5 | 32.1  52.8 / 8.0  55.3 / 10.4  44.8  2.2  22.9  0.8 | 35.6  60.6 / 7.3  63.7 / 10.4  53.3  2.7  27.3  0.9 | 32.2  51.0 / 4.8  61.4 / 15.2  46.2  2.1  28.0  1.4 |

^a^Values are means (SD) if not stated otherwise; ^b^One patient with GOLD category 1 COPD was included; ^c^Due to breathing problems. ^d^LABA not in fixed or combination therapy with ICS; ^e^ICS not in fixed or combination therapy with LABA.

BMI = body mass index; COPD = chronic obstructive pulmonary disease; FEV_1_ = forced expiratory volume in 1 second; GOLD = Global Initiative for Chronic Obstructive Lung Disease; ICS = inhaled corticosteroid; LABA = long-acting β_2_-agonist; SD = standard deviation.

**Supplementary Table 2** **Multivariate model for time-to-first exacerbation (all) and effect of covariates (completers only)**

| **Factor** | **Comparison^a^** | **N** | **HR** | **95% CI** | **p Value^b^** |
| --- | --- | --- | --- | --- | --- |
| **On-study treatment** | Tiotropium vs salmeterol | 3122/3021 | 0.81 | 0.74-0.88 | <0.0001 |
| **Baseline covariates** | | | | | |
| Sex | Female vs male | 1547/4596 | 1.30 | 1.18-1.43 | <0.0001 |
| COPD severity (GOLD category) | II^c^ vs III+IV | 3072/3071 | 0.83 | 0.76-0.91 | <0.0001 |
| **BMI, kg/m^2^** | | | | | 0.0057 |
|  | < 20 vs ≥ 20-< 25 | 432/2056 | 1.04 | 0.88-1.23 |  |
|  | < 20 vs ≥ 25-< 30 | 432/2126 | 1.17 | 0.99-1.39 |  |
|  | < 20 vs ≥ 30 | 432/1529 | 1.24 | 1.04-1.48 |  |
| **ICS use at baseline** | Yes vs no | 3183/2960 | 1.32 | 1.21-1.44 | <0.0001 |
| **COPD duration, y** | ≥ 6 vs < 6 | 3191/2940 | 1.15 | 1.06-1.26 | <0.0015 |
| **Smoking behavior, pack-years** | ≥ 36 vs < 36 | 3062/3081 | 1.08 | 0.99-1.18 | 0.0817 |
| **Antibiotic courses in prior year** | ≥ 2 vs < 2 | 1585/4500 | 1.39 | 1.26-1.54 | <0.0001 |
| **Corticosteroid courses in prior year** | ≥ 2 vs < 2 | 695/5065 | 1.31 | 1.15-1.49 | <0.0001 |

Time-to-first COPD exacerbation modelled by multivariate Cox regression with stepwise model selection; predictor had to be significant at the 0.25 level before being entered into the model and had to be significant at the 0.15 level to remain in the model; potential predictors originally also included smoking status (noncurrent smoker vs current smoker), concomitant diagnoses at baseline (0, 1, > 1) and age (< 50, ≥ 55 to < 65, ≥ 65 to < 75, ≥ 75).

^a^At least one exacerbation vs no exacerbation; ^b^Overall p value based on Wald’s chi-square test for optimal model; ^c^Includes one GOLD category 1 patient.

BMI = body mass index; CI = confidence interval; COPD = chronic obstructive pulmonary disease; GOLD = Global Initiative for Chronic Obstructive Lung Disease; HR = hazard ratio; ICS = inhaled corticosteroid.
